# Dose-dependent effects of chronic alcohol drinking on peripheral immune responses

**DOI:** 10.1038/s41598-019-44302-3

**Published:** 2019-05-24

**Authors:** Suhas Sureshchandra, Anthony Raus, Allen Jankeel, Brian Jin Kee Ligh, Nicole A. R. Walter, Natali Newman, Kathleen A. Grant, Ilhem Messaoudi

**Affiliations:** 1 Department of Molecular Biology and Biochemistry, University of California-Irvine, Irvine, CA 92697 USA; 2 Department of Biomedical Engineering, University of California-Irvine, 92697 Irvine, CA USA; 3 Oregon National Primate Research Center, Oregon Health & Science University, 97006 Beaverton, OR USA

**Keywords:** miRNA in immune cells, Monocytes and macrophages, Cytokines

## Abstract

It is well established that chronic heavy alcohol drinking (CHD) results in significant organ damage, increased susceptibility to infections, and poor outcomes following injury. In contrast, chronic moderate drinking (CMD) has been associated with improved cardiovascular health and immunity. These differential outcomes have been linked to alterations in both innate and adaptive branches of the immune system; however, the mechanisms remain poorly understood. To address this question, we determined the impact of chronic drinking on the transcriptional and functional responses of peripheral blood mononuclear cells (PBMC) collected from male rhesus macaques classified as CMD or CHD after 12 months of voluntary ethanol self-administration. Our analysis suggests that chronic alcohol drinking, regardless of dose alters resting transcriptomes of PBMC, with the largest impact seen in innate immune cells. These transcriptional changes are partially explained by alterations in microRNA profiles. Additionally, chronic alcohol drinking is associated with a dose dependent heightened inflammatory profiled at resting and following LPS stimulation. Moreover, we observed a dose-dependent shift in the kinetics of transcriptional responses to LPS. These findings may explain the dichotomy in clinical and immunological outcomes observed with moderate versus heavy alcohol drinking.

## Introduction

Observational studies in humans have reported strong associations between chronic heavy drinking (CHD) and significant organ damage as indicated by increased incidence of acute respiratory stress syndrome (ARDS)^1^, alcoholic liver disease (ALD)^2^, certain cancers^3–5^, cardiovascular diseases^6,7^, and sepsis^8^. Moreover, CHD results in increased susceptibility to pneumonia^9,10^, tuberculosis^11–13^, and faster progression of hepatitis C virus (HCV)^14^ and HIV infections^15^. Furthermore, individuals who engage in CHD exhibit higher rates of postoperative complications^16^, slower recovery from infection and trauma^16^, poor vaccine responses^17^, and wound healing^18^. In contrast, chronic moderate drinking (CMD) has been associated with decreased cardiovascular disease, improved insulin sensitivity, and decreased incidence of the common cold in humans^19–21^.

These studies suggest that chronic moderate and heavy drinking exert opposing effects on the human immune system; however, the mechanisms by which chronic drinking modulates immunity remain poorly understood. The earliest studies on CHD suggested that long-term drinking (several years to decades) is associated with reduction in T-cell numbers^22^, loss of naïve T-cells^22,23^, increased CD8+ T-cell activation and proliferation^24^, and increased serum immunoglobulin levels^25^. CHD also results in higher levels of circulating pro-inflammatory mediators TNFα, IL-1β, and IL-6^26^. Interpretation of these clinical studies are however confounded by age, erratic drinking patterns, sex, smoking status, use of recreational or illicit drugs, and nutritional deficiencies.

More recent *in vitro* studies where peripheral blood mononuclear cells (PBMC) isolated from healthy humans (not meeting the criteria of CHD) or monocytic cell lines were cultured with ethanol suggest that short (hours) and long-term (days) exposure have opposing effects on inflammatory responses of innate immune cells^27^. Specifically, while short-term exposure increased production of regulatory cytokines (e.g. IL-10) and decreased production of pro-inflammatory factors (TNFα and IL-6)^28–30^, long-term exposure heightened TNFα secretion following stimulation with toll-like receptor (TLR) 4 and 8 ligands^29,31,32^. However, these *in vitro* studies do not take into account the effects of ethanol’s metabolites and the pleiotropic impact of ethanol consumption on other immune cells, which can be modeled only using *in vivo* exposure.

The inhibitory effect of acute ethanol on production of pro-inflammatory cytokines in response to a variety of microbial compounds has been recapitulated using mouse models of acute ethanol exposure (reviewed in^27,33^). A handful of studies based in rodent models of chronic ethanol consumption have reported increased pathogen burden and impaired ability to clear *Listeria monocytogenes*^34^, *Mycobacterium tuberculosis*^35^, and influenza virus^36^. On the other hand, moderate alcohol exposure can improve microbial clearance and delay cutaneous hypersensitivity responses in rodents^37^. However, rodent studies employ gavage feeding, liquid diets, or intraperitoneal injections introducing other confounders such as nutritional status and nonvolitional stress for the animals. Finally, rodent models fail to recapitulate human patterns of light to moderate drinking.

To overcome these limitations, we used a rhesus macaque model of voluntary ethanol consumption established with a schedule-induced polydipsia procedure^38–40^. In this model, rhesus macaques are first induced to self-administer increasing daily doses of ethanol (0.5 g/kg/day to 1.5 g/kg/day over a 90-day period), then allowed “open access” to both water and 4% w/v ethanol for 22 h/day. During the open access phase to the protocol, the monkeys self-select their drinking status (CHD or CMD). This model presents a unique opportunity to study the impact of chronic voluntary moderate/heavy drinking on immunity in a highly translational outbred animal model without any overt tissue damage. We have leveraged this model to define the impact of chronic drinking on circulating and tissue resident immune cells^41–46^. Specifically, using this model, we have shown that CMD enhances while CHD attenuates vaccine responses^42,44,47^. More recently, we reported significant transcriptional changes in PBMC from female rhesus macaque following 12 months of CHD using RNA-Seq and concluded that the circulating innate immune cells bear the largest burden of chronic heavy drinking^46^. Therefore, in this study, we investigated the impact of both CHD and CMD on PBMC transcriptional profile and immune mediator production at resting and after stimulation with lipopolysaccharide (LPS) using samples obtained from three cohorts of male rhesus macaques that followed a standard protocol of daily open-access to 4% ethanol in water solution for over 12 months.

As previously reported with CHD females, we observe increased expression of genes in pathways regulating coagulation and inflammation with CHD, but not in CMD. Additionally, we observed a hyper-inflammatory phenotype both at resting and following LPS stimulation that was dose-dependent. Three-way comparison on RNA levels 16 hours post stimulation suggests that while transcriptional profile of PBMC from controls is suggestive of a sustained strong pro-inflammatory response, the transcriptional profile of PBMC from moderates indicates a return to baseline. PBMC from CHD animals, on the other hand, exhibited a transcriptional profile consistent with a regulatory phase of LPS response. These data strongly suggest a shift in inflammatory trajectory/timeline with chronic alcohol drinking.

## Results

### Chronic drinking alters transcriptional profiles of resting PBMC independent of ethanol dose

**Figure 1:**
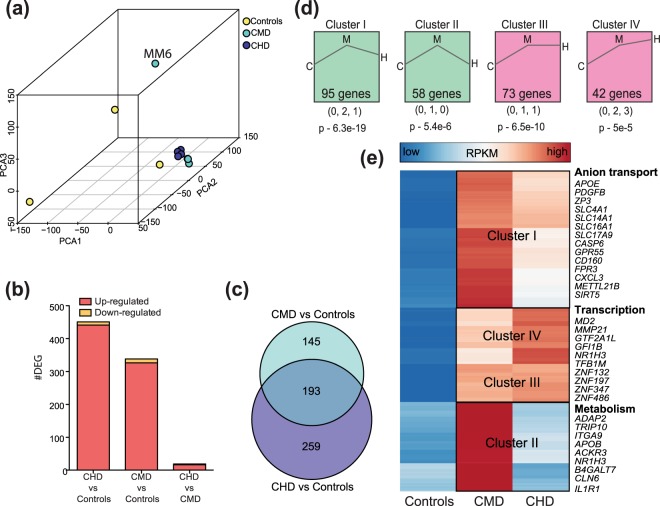
Chronic alcohol drinking alters PBMC gene expression independent of ethanol dose (**a**) 3D PCA of resting gene expression profiles of PBMC isolated from controls (n = 3), CMD (n = 4), and CHD (n = 4) animals. A potential outlier from CMD is highlighted but was retained in subsequent downstream analyses due to minimal changes in DEG profiles following its removal. (**b**) Bar graphs denoting number of differentially expressed genes (DEG) (protein coding and miRNA precursor) in CMD and CHD PBMC relative to control PBMC and each other. (**c**) Venn diagram describing overlap of DEG detected in CHD and CMD animals relative to controls. (**d**) Clusters of dose-dependent gene expression changes within the 597 genes dysregulated with chronic drinking (CMD and CHD combined) identified using STEM. Only statistically significant clusters (p < 0.05) are shown. Magnitudes of changes in controls (C), CMD (M), and CHD (H) are represented numerically below each representative cluster, with its statistical significance reported by STEM. (**e**) Heatmap of 268 ethanol sensitive DEG that demonstrated dose-dependent changes in expression (clusters I, II, III, and IV combined). Each row represents a gene with median expression level (RPKM) scaling from low (blue) to high (red). A subset of DEG within each cluster is highlighted.

To assess the overall impact of chronic drinking on peripheral immune cells, we performed RNA-Seq on PBMC isolated from three control animals and eight animals categorized as “chronic moderate drinkers” (CMD) (n = 4) or “chronic heavy drinkers” (CHD) (n = 4) at the end of 12 months of ethanol self-administration (Supplementary Fig. 1a–c). Principal Component Analysis (PCA) of PBMC transcriptomes demonstrate a substantial effect of chronic drinking, where transcriptional profiles of samples obtained from CMD and CHD animals segregate away from those of control animals (Fig. 1a). Furthermore, alcohol is a strong determinant of resting PBMC transcriptome, demonstrated by reduced within group variability in gene expression profiles (Supplementary Fig. 2a). Differential gene expression analysis using edgeR (Supplementary Table 1) identified 452 and 338 differentially expressed genes (DEG; FDR ≤ 0.05) that were mostly upregulated with ethanol consumption in both CHD (410 of 452) and CMD (302 of 338) groups respectively relative to the control group (Fig. 1b) with a significant overlap between these two DEG subsets (p < 0.0001, hypergeometric test) (Fig. 1c). Limited gene expression changes were detected between the CMD and CHD groups, indicating that ethanol consumption is the major driver of these transcriptional changes (Fig. 1b). These differences were observed in the absence of any differences in immune cell frequencies among the three groups^43^.

DEG (annotated protein coding and microRNA genes) detected only in CHD group enriched to gene ontology (GO) terms associated with inflammation (e.g., *CRP*, *ALOX5*, *TF*, *C1S*, and *C9*), signaling, and blood homeostasis (e.g. fibrinogen genes *FBA*, *FGB*, and *FGG* as well as wound healing factors *TBXA2R*, *SERPING1*, and *SERPINB2*) (Supplementary Fig. 2b). Additional analyses of protein-protein interactions indicate that DEG detected only in CHD group play a role in membrane trafficking, mitochondrial protein synthesis, as well as GPCR and TLR signaling pathways (Supplementary Fig. 2c). The DEG detected only in the CMD group were primarily involved in cellular chemical homeostasis (*SNCA*, *SLC4A1*, *SLC4A10*, *SLC35G1*), chemotaxis (*FPR3*, *C5AR2*, *GPR18*), and cytokine signaling (*CD200R1*, *NR1H3*, *ECM1*) (Supplementary Fig. 2d). The 193 DEG detected in both CMD and CHD groups relative to controls (Fig. 1c) enriched to GO terms associated primarily with metabolic pathways (Supplementary Fig. 2e).

In order to explore dose-dependent changes in gene expression, we next combined DEG detected in CHD and CMD relative to controls and modeled expression profiles of these genes using Short Time-series Expression Miner (STEM)^48^, replacing time with ethanol dose as the independent variable. This analysis identified four significant clusters (p < 0.05) (Fig. 1d and Supplementary Fig. 2f) with distinct patterns of dose dependence. For example, expression levels of genes in cluster I increased with chronic drinking but reached higher levels in the CMD group (Fig. 1d and Supplement Fig. 2f). These DEG play a role in anion transport and cellular signaling (Fig. 1e). Cluster II contained DEG the expression of which only increased with CMD (Fig. 1d and Supplement Fig. 2f). These DEG are important for cellular metabolism and ECM-receptor interaction (*IL1R*, *ITGA9*, *FGG*) (Fig. 1e). Finally, expression of DEG in cluster III increased with CMD and plateaued, while expression of DEG in cluster IV progressively increased with ethanol consumption. DEG in both of these clusters were involved in transcriptional regulation (several zinc finger proteins) and myeloid cell activation (*MD2*, *NR1H3*, and *MMP21*) (Fig. 1e).

To get a better understanding of the cellular sources of the DEG detected, we next used the ImmGen database^49^, which visualizes the distribution of genes across immune cell populations to infer the source of the gene expression changes detected. In agreement with our previous study in CHD females^46^, the majority of the DEG detected with both CMD and CHD are highly expressed by monocytes and dendritic cells (DCs) (Supplementary Fig. 3a,b).

### CMD and CHD alter microRNA (miRNA) profiles of PBMC

Analysis of RNA-Seq data indicated changes in some microRNA (miRNA) precursors with both CHD and CMD. To gain a deeper understanding of the impact of drinking on miRNA expression patterns, we next profiled all cellular mature miRNA using an additional sequencing experiment (Supplementary Fig. 1c, Supplementary Table 2) (n = 4/group). We identified 534 macaque miRNAs with a median expression level (RPKM) > 1. To assess the role of miRNA in PBMC from rhesus macaques, we identified 8,856 validated gene targets for the 100 most highly expressed miRNAs from control animals using miRNet. Functional enrichment of these genes indicates that they play a role in pathogen sensing, response to infections, and adaptive immune signaling (Supplementary Fig. 4a).

**Figure 2:**
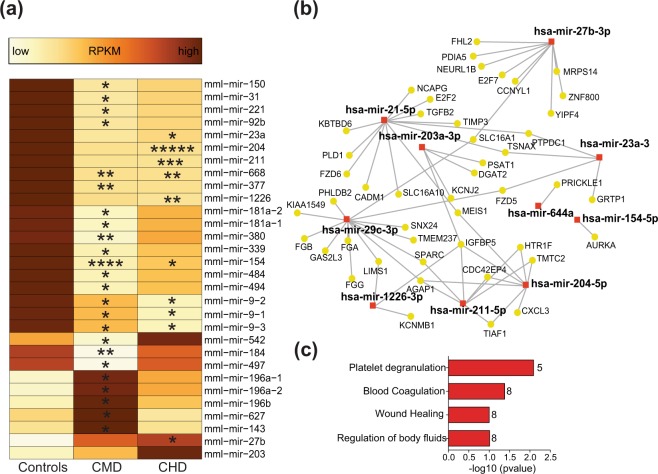
Chronic alcohol drinking alters microRNA profile of PBMC. (**a**) Heatmap showing median RPKM of ethanol sensitive mature microRNAs from controls (n = 4), CMD (n = 4), and CHD (n = 4) animals detected using small RNA sequencing. Colors represent median transcript levels (RPKM) scaled from low (yellow) to high (brown). Asterisks indicate levels of statistical significance relative to controls: *p ≤ 0.05; **p ≤ 0.01; ***p ≤ 0.001; ****p ≤ 0.001; ****p ≤ 0.0001. (**b**) Network of miRNAs dysregulated with CHD (red squares) and their validated mRNA targets that are dysregulated with CHD (yellow circles) built using miRNet. (**c**) Functional enrichment of the targets highlighted in (**b**) predicted using Reactome and GO database in miRNet. (P-values were derived from hypergeometric tests). Numbers next to the bar denote genes mapping to each functional enrichment term.

Surprisingly, only 11 differentially expressed miRNA genes, which were mostly downregulated with drinking (9 of 11), were detected with CHD (Fig. 2a). Of the 452 mRNA DEG detected with CHD, 46 are validated targets of these differentially expressed mature and precursor miRNAs (Fig. 2b). Moreover, these 46 DEG play roles in regulation of body fluids, coagulation, and wound healing (Fig. 2c). On the other hand, CMD was associated with 24 differentially expressed miRNA, which were also mostly downregulated with drinking (19 of 24). These miRNA (Fig. 2a) were associated with 60 validated gene targets within the 338 mRNA DEG with CMD (Supplementary Fig. 4b). Finally, different microRNA were upregulated with CMD (miR-196, miR-143, and miR-627) and CHD (miR-27b and miR-203).

### Chronic drinking alters the immune response to LPS in a dose-dependent manner

**Figure 3:**
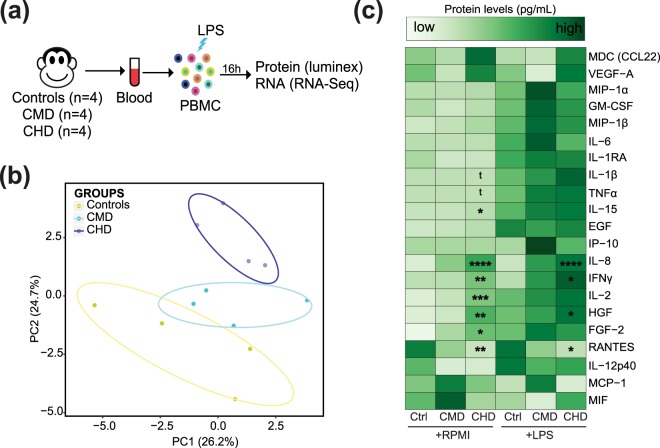
Chronic alcohol drinking alters immune responses to LPS. (**a**) Experimental design: PBMC from controls (n = 4), CMD (n = 4), and CHD (n = 4) animals were cultured in the presence or absence of LPS for 16 h. Supernatants were collected for immune mediator measurements using multiplex bead assay and the cell pellets were used for RNA extraction and RNA-Seq. (**b**) Principal Component Analysis (PCA) of overall immune mediator profiles (21 analytes detected above limits of detection) produced in the absence of LPS stimulation. Each circle represents a biological sample. (**c**) Heatmap of median secreted analyte concentrations (pg/mL) before and after LPS stimulation. Dose associated linear increases or decreases in secreted levels both before and after stimulation were independently tested using linear regression analysis. Analytes with statistically significant and trending linear association with ethanol consumption dose are marked (t – p = 0.05; *p ≤ 0.05; **p ≤ 0.01; ***p ≤ 0.001; ****p ≤ 0.001, F-tests). Protein concentrations are scaled from low (light green) to high (dark green).

Given that the majority of transcriptional changes are predicted to originate from monocytes and DCs (Supplementary Fig. 3a,b), we next determined if chronic drinking results in altered responses to TLR ligands. PBMC from control (n = 4), CMD (n = 4), and CHD (n = 4) animals were cultured for 16 hours in the presence or absence of TLR4 agonist LPS (Fig. 3a). We then measured differences in immune mediator production using a multiplex bead array and changes in gene expression using RNA-Seq (Fig. 3a). First, we assessed the impact of chronic drinking on spontaneous immune mediator production using principal component analyses (PCA), which revealed distinct resting cytokine profiles (Fig. 3b) (Supplementary Table 3). Additionally, we measured the relationship between secreted levels of analytes and ethanol dose using linear regression (Fig. 3c and Supplementary Fig. 5a). This analysis revealed a dose dependent increase in spontaneous production of inflammatory cytokines IFNγ (p = 3e-3), IL-15 (p = 2e-2), IL-2 (p = 5e-4), TNFα (p = 0.05), and IL-1β (p = 0.05); growth factors HGF (p = 2e-3) and FGF-2 (p = 0.01); and chemokines IL-8 (p = 1e-4) (Fig. 3c and Supplementary Fig. 5a). Interestingly, we observed a dose-dependent reduction in spontaneously secreted levels of RANTES (p = 4e-3) (Fig. 3c and Supplementary Fig. 5c).

Following LPS stimulation, we observed a dose-dependent increase in IFNγ (p = 9e-3), IL-8 (p < 0.0001), and HGF (p = 0.03) secretion with chronic drinking (Fig. 3c and Supplementary Fig. 5b). Additionally, we observed a linear trend in post LPS IL-1β induction with ethanol dose (p = 0.1) (Supplementary Fig. 5c). As described above for spontaneous production, we noted a dose-dependent reduction in post LPS induction of RANTES (p = 0.02) as well as MIP-1β (p = 0.01) (Fig. 3c and Supplementary Fig. 5c).

### Chronic drinking alters transcriptional responses to LPS in a dose-dependent manner

**Figure 4:**
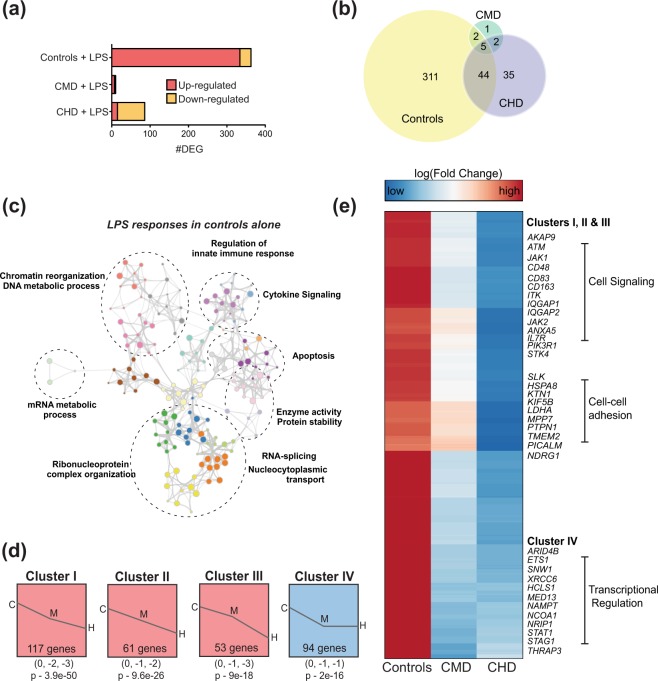
CMD and CHD exhibit distinct transcriptional responses to LPS in PBMC. (**a**) Bar graphs of protein coding DEG detected at 16 hours post LPS stimulation in PBMC from controls (n = 4), CMD (n = 4), and CHD (n = 4) animals. (**b**) 3-way Venn illustrating the overlap of DEG detected following LPS stimulation in controls, CMD and CHD. (**c**) Network of GO terms to which DEG detected in response to LPS in control group only (311 genes) generated using Metascape. Each bubble represents a GO term and clusters of similar GO terms are placed in bubbles delineated with dashed lines and labeled with the most statistically significant GO term. Size of the bubble represents number of genes mapping to the GO term and gray line indicate strength of association between different GO terms. (**d**) Modeling dose-dependent transcriptional responses to LPS using fold changes (log scale) of 400 genes identified in (**b**) using STEM. Only statistically significant clusters (p < 0.05) are shown. Magnitudes of fold changes in controls (C), CMD (M), and CHD (H) are represented numerically below each representative cluster, with its statistical significance reported by STEM. (**e**) Clustered heatmap of 325 genes that demonstrate dose dependent transcriptional responses to LPS (clusters I, II, III, and IV combined). Each row represents a gene with fold change (log scale) relative to unstimulated controls scaling from low (blue) to high (red). Subsets of genes within each cluster are highlighted.

We next determined if alterations in the response to LPS with chronic drinking were recapitulated at the transcriptional level (Supplementary Table 4). In contrast to the control group where we observed a strong induction of a gene expression at 16 hours (362 DEG), we detected limited transcriptional changes in the CMD group (10 DEG), and down-regulation of 86 DEG in CHD (Fig. 4a), 44 of which were also detected in controls (Fig. 4b). DEG detected in control group only (Fig. 4b) played a role in canonical inflammatory response to LPS as indicated by functional enrichment to GO terms “innate immune response” and “cytokine signaling” as well as regulatory modules “mRNA metabolic process” and “chromatin reorganization” (Fig. 4c). Next, we explored potential dose dependent changes in gene expression changes. STEM analysis of all the 390 LPS-responsive DEG (Fig. 4b) identified four distinct but significant clusters (p < 0.001) (Fig. 4d). DEG in clusters I, II, and III displayed ethanol dose-dependent changes (Fig. 4d and Supplement Fig. 6). DEG in these 3 clusters played a role in inflammation (*CD83*, *CD163*), signaling (*JAK1*, *STK4*, *PIK3R1*) and cell adhesion (*TMEM2*, *PTPN1*, *KTN1*, *SLK*) (Fig. 4e). DEG in cluster IV were down regulated with drinking regardless of dose and are involved in transcriptional regulation (Fig. 4d,e).

**Figure 5:**
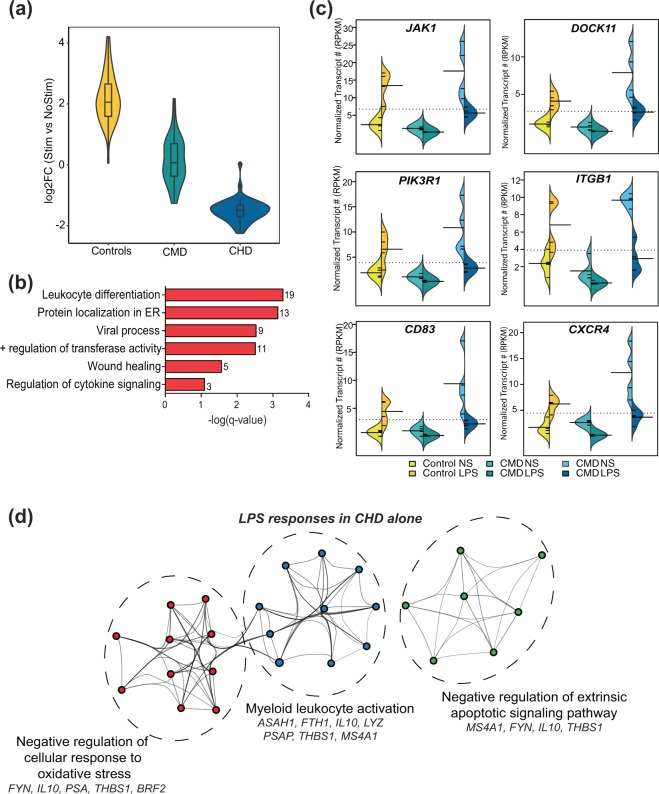
Dose-dependent transcriptional responses to LPS with chronic alcohol drinking. (**a**) Violin plot representing log_2_ Fold Change of the 44 DEG detected in both CHD and control PBMC following LPS stimulation. (**b**) Functional enrichment of the 44 DEG detected in both controls and CHD groups. Numbers next to the bar represent numbers of genes mapping to each term. (**c**) Bean plots of RPKM levels of select DEG detected in both control and CHD animals. (**d**) Network depicting functional enrichment of the DEG detec ted exclusively dysregulated in CHD following LPS stimulation identified using Metascape. Each colored bubble represents a GO term clustered within a dotted circle with annotations and genes mapping to the terms.

Of the 44 DEG detected in both controls and CHD (Fig. 4b), 43 showed opposite directional changes (upregulated in controls but downregulated in CHD), and no change in expression in CMD (Fig. 5a). These genes play roles in cytokine signaling, leukocyte differentiation, and wound healing (Fig. 5b). More importantly, this list includes genes involved in sustaining cytokine production (*JAK1*, *DOCK11*, and *PIK3R1*) and cell surface receptors involved in activation (*CD83*), adhesion (*ITGB1* which encodes VCAM1*)*, and chemoattraction (*CXCR4*) (Fig. 5c). The largely downregulated DEG exclusively detected with CHD (Fig. 4a) played a role in myeloid leukocyte activation and regulation of stress response (Fig. 5d).

## Discussion

In this study, we used a macaque model of voluntary ethanol self-administration to investigate the impact of CMD and CHD on the PBMC transcriptional profile and its response to LPS. Specifically, PBMC isolated from male rhesus macaques that were classified as controls or categorized as CMD or CHD based on their patterns of ethanol self-administration over a 12-month period were subjected to RNA-Seq and Luminex analysis before and after LPS stimulation. While there is a significant overlap between gene expression changes induced by CMD and CHD, transcriptional changes exclusively detected with CHD enriched to blood coagulation, immune signaling and wound healing pathways. This observation is in line with the increased susceptibility to cardiovascular disease^6,7^ and impaired wound healing^16,18,50^ observed in subjects with alcohol use disorder. CMD on the other hand triggers activation of cellular metabolic pathways with dose-associated increases in expression of genes involved in transcriptional regulation and myeloid cell activation. To our knowledge, this is the first report of transcriptional changes in resting immune cells with moderate drinking. Furthermore, profiling miRNA repertoire further indicates that the pro-inflammatory impact of drinking is significant only in CHD and not in CMD.

As we recently reported for gene expression changes of resting PBMC isolated from CHD female rhesus macaques^46^, additional mining of data using online databases suggested that that a majority of these transcriptional triggers in PBMC from both CMD and CHD originate from DCs and monocytes. Moreover, gene expression changes reported in this study of CHD males enrich to similar biological processes, notably immune signaling and coagulation pathways, as those reported for PBMC from CHD females^46^. In both cases, these differences were observed in the absence of changes in frequencies of major circulating immune cells^43,46^ suggesting potential immune reprogramming with both CMD and CHD, most likely in innate immune cells. These observations are in line with previous reports of significant changes in innate immune branch within short period of exposure to ethanol while changes in adaptive branch are often only detected after decades of alcohol dependence in humans or >6 months of chronic consumption of high concentration of ethanol solution in rodents (equivalent to 20 human years)^44^.

The observation that ethanol sensitive gene expression changes enrich to pathways that regulate platelet degranulation and coagulation is consistent with the fact that alcohol use disorder interferes with several aspects of the blood clotting system, causing abnormally low platelet numbers in the blood, impaired platelet function, and diminished fibrinolysis^51^. Sex-specific differences in the impact of ethanol consumption on immune cells transcriptional profile may be attributed to differences in signaling by sex steroids. It is, however, important to note that some of the females from our previous study consumed higher average levels of ethanol over the 12 months of open access (range from 3.29–5.17 g/kg/day) than the CHD males in this study (range from 3.01–4.21 g/kg/day). Therefore, at this point we are unable to tease the impact of sex from those of ethanol dose on immunity.

Innate immune cells may be more sensitive to the impact of ethanol consumption due to their short half-life and rapid turnover production from the bone marrow. In contrast, lymphocytes are primarily produced during fetal development and are very long-lived. However, it is currently unclear whether this impact is mediated by ethanol metabolites acting on circulating myeloid cells or if ethanol reprograms the myeloid precursors in the bone marrow during their lineage development. The involvement of both mechanisms is highly likely since chronic binge drinking has been shown to impair activation of granulocyte precursor cells in response to bacteremia in mice^52^.

Given that both CMD and CHD exert a dramatic impact on myeloid cells, which play a critical role in wound healing and battling infections; we investigated changes in response to LPS as a model bacterial antigen. Previous *ex vivo* and *in vitro* studies have reported an exposure dependent change in the monocyte responses to LPS where acute exposure to ethanol is associated with dampened monocyte responses to LPS and prolonged exposure is associated with enhanced LPS response (reviewed in^44^). However, these studies stimulated human PBMC or THP1/RAW cell lines with LPS for various durations in the presence of different amounts of ethanol and measured canonical LPS inducible cytokines as readouts. Furthermore, the impact of moderate and heavy drinking on LPS inducible transcriptional response was unknown.

At the protein level, linear regression analyses suggest strong dose dependence in the secretion of several pro-inflammatory factors and growth factors both under resting conditions (TNFα, IL-1β, IFNγ, IL-2, FGF2, and HGF) and post LPS exposure (IL-8, RANTES, HGF, and IFNγ). These findings are in agreement with recent clinical studies that showed greater inflammatory mediator production by alveolar macrophages and PBMC obtained from human excessive/heavy alcohol consumers in response to LPS and lipoteichoic acid (LTA)^53^. While post-LPS production of cytokines *ex vivo* is a measure of immunocompetence, spontaneous production of cytokines has been previously linked to a state of *in vivo* activation^54^. Indeed, in line with this hypothesis, a recent report in humans suggested that the ethanol dose consumed positively correlated with serum markers of monocyte activation^55^, providing a potential explanation for heighted pro-inflammatory responses to LPS in CHD. Interestingly, our analysis revealed dose-associated drop in spontaneously produced RANTES and post LPS induction of MIP-1β. However, given that these *in vitro* experiments involved mixed populations of immune cells, it is hard to attribute this trend to reduced production or enhanced signaling of chemokine factors.

Several potential mechanisms could explain a state of heightened immune cell activation following chronic ethanol consumption. Alcohol has been shown to disrupt the gut intestinal epithelial barrier permeability^56,57^, leaking microbial antigens and endotoxins into circulation in acute binge drinking models^58^. In humans, long term drinking has been associated with higher serum endotoxin levels, which increase in a dose-dependent manner^55^. Similarly, we have recently reported a dose-dependent increased in the levels of circulating IgM-bound endotoxin levels in the animals used in this study^45^. It is, however, still unclear if ethanol-induced leakage of microbial antigens reprograms the bone marrow to influence hematopoiesis or acts on circulating immune cells. Aberrant activation of myeloid precursor cells would suggest that the effects of heavy drinking on the immune system are both long-term and irreversible.

A hyperactive resting state may interfere with the generation of appropriate responses to microbial antigens. To begin to address this question, we investigated the transcriptional response to LPS stimulation. RNA-Seq measurements in PBMC from control animals at 16 hours post stimulation indicate activation of canonical inflammatory pathways associated with an LPS response such as cytokine signaling and innate immune response; and regulatory mechanisms such as mRNA catabolism, translation, and apoptosis. In contrast, responses in CHD animals indicate down-regulation of several genes involved in leukocyte differentiation, wound healing, and cytokine signaling. These observations are suggestive of a resolution phase. This scenario is corroborated by up-regulation of *IL10*, essential for a strong anti-inflammatory response^59^, in CHD animals only (Fig. 5d). It is possible that a heightened activation state of resting PBMC from CHD prevents them from further up-regulating expression of canonical pathways essential for the LPS response, suggestive of an immune tolerant phenotype.

Alterations in responses of PBMC from CHD to LPS can also be attributed to epigenetic changes. We have previously shown that ethanol sensitive miRNAs explain vaccine induced gene expression changes in PBMC^44^. In this study, we report changes in expression of mature and precursor miRNA molecules, which have validated roles in LPS signaling (miR-21, miR-29, miR-211, and miR-204)^60,61^. miR-204, for example, is a negative regulator of TLR4 signaling^62^ through its target *JAK2*. Decreased expression of miR-204 with CHD correlates with enhanced pro-inflammatory phenotype observed with CHD post LPS^63^. However, the small number of miRNA detected in this study strongly suggest that these mechanisms of post-transcriptional regulation may not represent the primary mode by which ethanol alters immune cell functions, at least following *ex vivo* LPS stimulation. In addition to post-transcriptional mechanisms, ethanol has been shown to impact transcriptional mechanisms in the CNS at the genomic level, such as chromatin remodeling^64–66^ and DNA methylation^67,68^. Future work will focus on identifying changes in chromatin accessibility; patterns of histone methylation and acetylation in purified monocytes and DC subsets to better understand ethanol’s impact on immune function.

Overall, our data suggest that moderate and heavy alcohol drinking exert distinct transcriptional and post-transcriptional changes in circulating immune cells. Furthermore, alcohol drinking impacts inflammatory responses to microbial products in a dose-dependent manner. To our knowledge, this is the first comprehensive characterization of alcohol dose-dependent changes in circulating immune cells in the absence of overt organ damage. Given the significant similarity between human and nonhuman primate physiologic and immunologic systems, these findings have a high translational value.

## Methods

### Sample collection

Blood samples were collected from 17 male rhesus macaques (average age 6.4 yrs at sample collection), with five animals serving as controls and eight classified as chronic moderate drinkers (CMD) and four as chronic heavy drinkers (CHD) after 12 months of ethanol self-administration through the Monkey Alcohol Tissue Research Resource (www.matrr.com; Supplementary Fig. 1a,b). These cohorts of animals were previously described in detail^38,69^ and leveraged in several immunological studies^42,44,45^. Differentials from whole blood were obtained using a complete blood count machine calibrated for rhesus blood. Peripheral Blood Mononuclear Cells (PBMC) were isolated by centrifugation over histopaque (Sigma, St Louis, MO) as per manufacturer’s protocol and cryopreserved in FetalPlex™ Animal Serum Complex/DMSO until they could be analyzed as a batch. The average daily ethanol dosage for each animal ranged from 1.5 g/kg to 4.2 g/kg as outlined in Table 1. Animals with average daily ethanol consumption <3 g/kg were considered chronic moderate drinkers (CMD) whereas ones with daily consumption >3 g/kg were considered chronic heavy drinkers (CMD). Daily ethanol consumption of individual animals is shown in Supplementary Fig. 1b. A subset of samples was used for each of the analyses as shown in Table 1.

**Table 1:** Summary of animals used in each experiment and drinking behavior in this study.

Sample ID	MATRR ID	12 month average ethanol (g/kg/day)	Group	RNA-Seq	Micro RNA-Seq	LPS Stim
MC1	10093	0	Control	X	X	X
MC2	10185	0	Control	X		
MC3	10220	0	Control	X	X	X
MC4	10095	0	Control		X	X
MC5	10184	0	Control		X	X
MM1	10211	2.1	CMD	X		
MM2	10086	2.28	CMD		X	X
MM3	10089	1.95	CMD	X		
MM4	10092	1.85	CMD	X		
MM5	10085	2.07	CMD		X	X
MM6	10084	1.82	CMD	X		
MM7	10087	2.34	CMD		X	X
MM8	10210	1.54	CMD		X	X
MH1	10097	3.01	CHD	X	X	X
MH2	10091	3.09	CHD	X	X	X
MH3	10098	3.32	CHD	X	X	X
MH4	10214	4.21	CHD	X	X	X

### LPS Stimulation assay

A million freshly thawed PBMC were cultured in RPMI supplemented with 10% FBS with or without 100 ng/mL LPS (TLR4 ligand, *E*. *coli* 055:B5; Invivogen, San Diego CA) for 16 hours in 96-well tissue culture plates at 37 C in a 5% CO_2_ environment. Plates were spun down: supernatants were used to measure production of immune mediators and cell pellets were resuspended in Qiazol (Qiagen, Valencia CA) for RNA extraction. Both cells and supernatants were stored at −80 °C until they could be processed as a batch.

### RNA extraction and RNA-Seq library preparation

Total RNA was isolated from PBMC using the mRNeasy kit (Qiagen, Valencia CA) and quality assessed using Agilent 2100 Bioanalyzer. Following ribosomal RNA depletion using Ribo-Gone rRNA removal kit (Clontech, Mountain View CA), libraries were generated using SMARTer Stranded RNA-Seq kit. Briefly, rRNA depleted RNA was fragmented, converted to double-stranded cDNA and ligated to adapters. The roughly 300bp-long fragments were then amplified by PCR and selected by size exclusion. Libraries were multiplexed and following quality control for size, quality, and concentrations, were sequenced on single-end mode to an average depth of 20 million 100 bp reads.

### Small RNA library preparation

Small RNA libraries were generated from total RNA as starting material using QIAseq miRNA Library kit (Qiagen, Valencia CA) per manufacturer’s instructions. Total RNA (10 ng) was 3′ (1:5 dilution) and 5′ adapter (1:2.5) ligated using miRNA specific adapters. Following reverse transcription (adapters 1:5 dilution), cDNA was size selected using QIASeq miRNA NGS beads and PCR amplified for 19 cycles. Following final cleanups, quality and concentrations of the libraries were measured using Bioanalyzer 2100 (Agilent, Santa Clara CA), and size selected to obtain libraries with a target peak 173 bp (average range 145–205 bp) using Pippin Prep (Sage Biosciences, Beverly MA). Final peak sizes and concentration of libraries was verified on the Bioanalyzer. Libraries were sequenced on NextSeq 2500 yielding libraries of 10 million reads each of 75 bp sequenced on single-end mode.

### Bioinformatics analysis

RNA-Seq reads were quality checked using FASTQC, adapter and quality trimmed using TrimGalore, retaining reads at least 35 bp long. Reads were aligned to *Macaca mulatta* genome (Mmul_8.0.1) based on annotations available on ENSEMBL (Mmul_8.0.1.92) using TopHat^70^ internally running Bowtie2. Aligned reads were counted gene-wise using GenomicRanges^71^, counting reads in a strand-specific manner. Read counts were normalized using RPKM method for generation of PCA and heatmaps. Pair-wise distances between samples within each group were calculated on raw counts following regularized log transformation using rlog and dist functions in DESeq. 2. Genes with average raw counts (across all samples) <5 were removed to improve the statistical power of differentially expressed genes. Raw counts were used to identify differentially expressed genes (DEG) using edgeR^72^. DEG are defined as those with at least two-fold change in expression and an FDR controlled at 5%. Only DEG with known macaque gene annotations (protein coding and miRNA precursors) and a minimum median normalized transcript expression (RPKM) of at least 1 were included in all downstream analyses. Functional enrichment of gene expression changes in resting cells was performed using Metascape^73^ and InnateDB. Networks of functional enrichment terms were generated using Metascape^73^ and visualized in Cytoscape^74^.

Small RNA reads were trimmed to 15–30 bp range using TrimGalore (www.bioinformatics.babraham.ac.uk/projects/trim_galore/) and aligned to the macaque genome using bowtie2 (-k 50 –very-sensitive-local) using miRNA annotations available on ENSEMBL (Mmul_8.0.1.92). Read counting and differential gene expression was performed as described for RNA-Seq with the exception that genes with p < 0.05 were defined as differentially expressed. For all downstream analysis of small RNA regulation, we combined this list of DEG with differentially expressed miRNA precursors detected from bulk RNA-Seq.

### ImmGen analysis

Most probable cellular sources of DEG were identified using ImmGen database’s (www.immgen.org) “My GeneSet” application, excluding immune cells not typically observed in PBMC. Genes lowly expressed across all cell types were excluded in this analysis.

### Analysis of dose-dependent changes using STEM

Dose-based clustering of genes was performed using Short Time-series Expression Miner (STEM) (http://www.cs.cmu.edu/~jernst/stem/) using ethanol consumed (g/kg/day) as a continuous variable modeling median normalized transcript levels (RPKM) for every gene as the measured variable. DEG detected following CHD versus controls and CMD versus controls were included in these analyses. Only statistically significant clusters (p < 0.05) were retained for downstream interpretation. To measure group differences in identified clusters, gene specific fold change/RPKM were tested for differences using repeated measures one-way ANOVA followed by Holm-Sidak’s multiple comparisons test.

### mRNA-miRNA Integration

Experimentally validated gene targets of differentially expressed miRNA were identified using miRNet^75^. Only direct mRNA-miRNA interactions were retained and mRNA differentially expressed from RNA-Seq experiment were highlighted using BatchHighlighter option. Functional enrichment of the highlighted genes was tested using miRNet’s inbuilt hypergeometric tests (p < 0.05) against GO and Reactome databases.

### Luminex assay

Circulating immune mediators were measured using nonhuman primate Cytokine/Chemokine/GF (eBioscience, San Diego CA) 29-plex panel measuring levels of cytokines (IFNγ, IL-1β, IL-2, IL4, IL-5, IL-6, IL-12, IL-15, IL-17, TNFα, IL-1RA, IL-10, and MIF), chemokines (MCP-1, MIP-1α, MIP-1β, RANTES, Eotaxin, MDC, IL-8, MIG, and I-TAC), and growth factors (EGF, FGF, G-CSF, GM-CSF, HGF, and VEGF-A). Standard curves were generated using 5-parameter logistic regression using the xPONENT™ software provided with the MAGPIX instrument (Luminex, Austin TX). Dose-dependent secretion and fold induction were modeled based on g/kg/day ethanol consumed and tested for linear fit using regression analysis in Prism 8 (GraphPad, San Diego CA). Metrics for goodness of fit (r^2^) is reported in each figure. P-values were calculated using F-tests testing if the slope was significantly non-zero.

### Data visualization

Graphs were generated using several packages in R – dendrograms (DESeq2), venn diagrams (VennDiagram), clustered heatmaps (gplots and lattice), PCA (ggbiplot), 3D-PCA (scatterplot3D), violin plots (ggplot2), beanplots (beanplot). Functional enrichment of overlapping GO terms and protein-protein interactions were visually presented as network images generated in Metascape and Cytoscape. Functional enrichment of standalone GO terms was presented as bar graphs generated in Prism 8. mRNA-microRNA networks were generated in miRNet.

### Ethics approval

This study was performed in strict accordance with the recommendations made in the Guide for Care and Use of Laboratory Animals of the National Institutes of Health, the Office of Animal Welfare and the United States Department of Agriculture. The ONPRC Institutional Animal Care and Use Committee approved all animal work.

## Supplementary information

Supplementary Figures

Supplementary Table 1

Supplementary Table 2

Supplementary Table 3

Supplementary Table 4

## Data Availability

All small RNA and mRNA sequencing data have been deposited in NCBI’s Sequence Read Archive under project accession number PRJNA523863.
