# Supplementary material for: Dose-dependent effects of chronic alcohol drinking on peripheral immune responses
Source: Sci Rep. 2019 May 24;9:7847. doi: 10.1038/s41598-019-44302-3 (PMC6534547; doi:10.1038/s41598-019-44302-3)
Supplement: Supplementary file 1 — Supplementary Figures [file 41598_2019_44302_MOESM1_ESM.pdf]

Dose-dependent effects of chronic alcohol drinking on peripheral immune responses

Suhas Sureshchandra[1], Anthony Raus[1], Allen Jankeel[1], Brian Jin Kee Ligh[2], Nikki Walter[3], Natali Newman[3], Kathleen A. Grant[3], Ilhem Messaoudi1,[3]*

[1]Department of Molecular Biology and Biochemistry, University of California-Irvine, Irvine, CA 92697, USA
[2]Department of Biomedical Engineering, University of California-Irvine, Irvine, CA 92697, USA
[3]Oregon National Primate Research Center, Oregon Health & Science University, Beaverton, OR 97006, USA

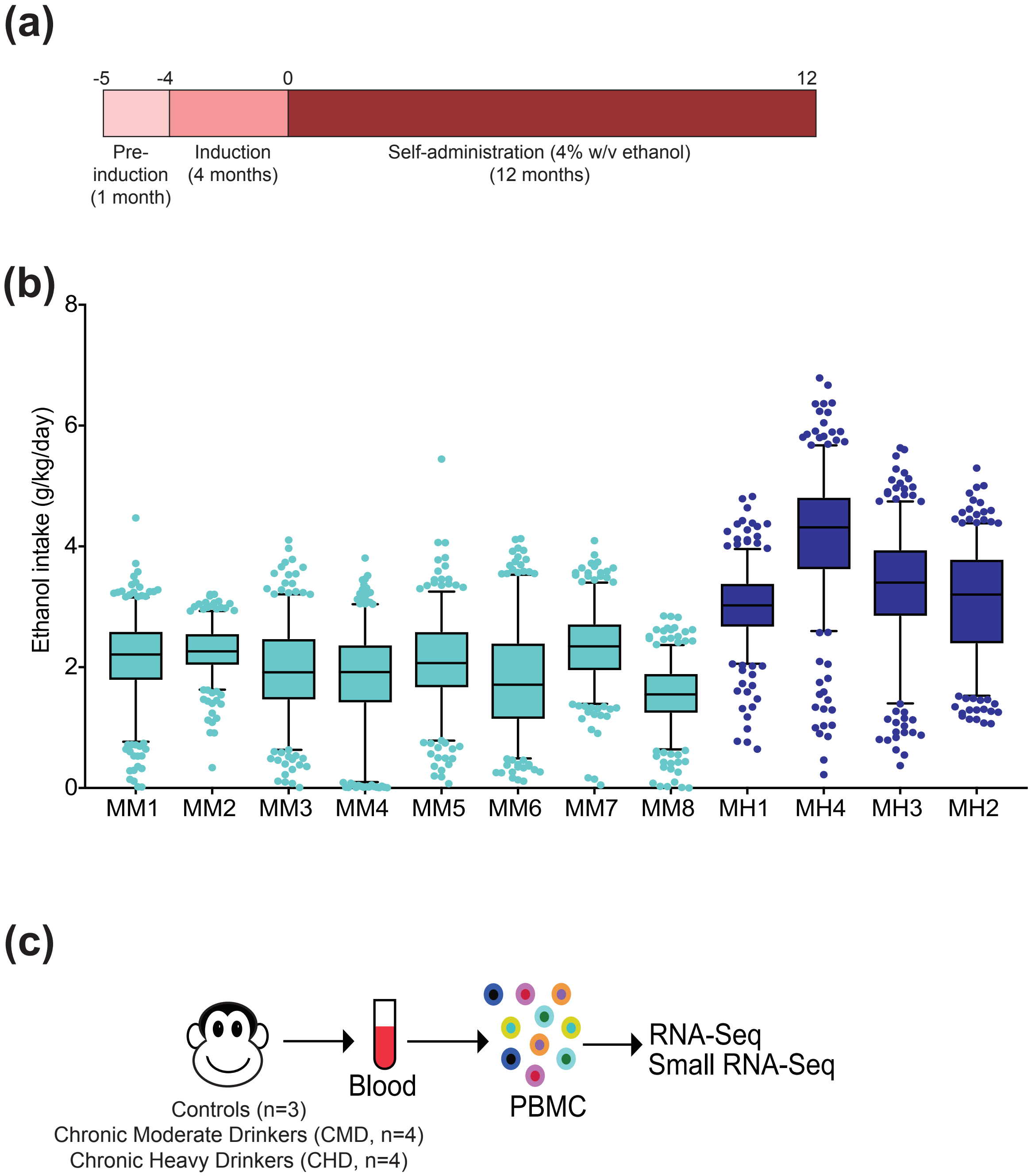

Supplementary Figure 1: Study design (a) Schematic of the timeline for the experimental rhesus macaque model of chronic ethanol self-administration. (b) Box and whiskers plot denoting overall drinking patterns of the animals used in this study. Boxes represent the first and the third quartile of g/kg ethanol intake from every single drinking day. The whiskers represent 5-95 percentile of the data. Animals with average daily ethanol consumption < 3g/kg were considered moderates (CMD) whereas ones with g/kg >3 were considered heavy drinkers (CHD). (c) Design and experimental readouts for RNA-Seq experiments measuring impact of CMD and CHD on basal PBMC profiles.

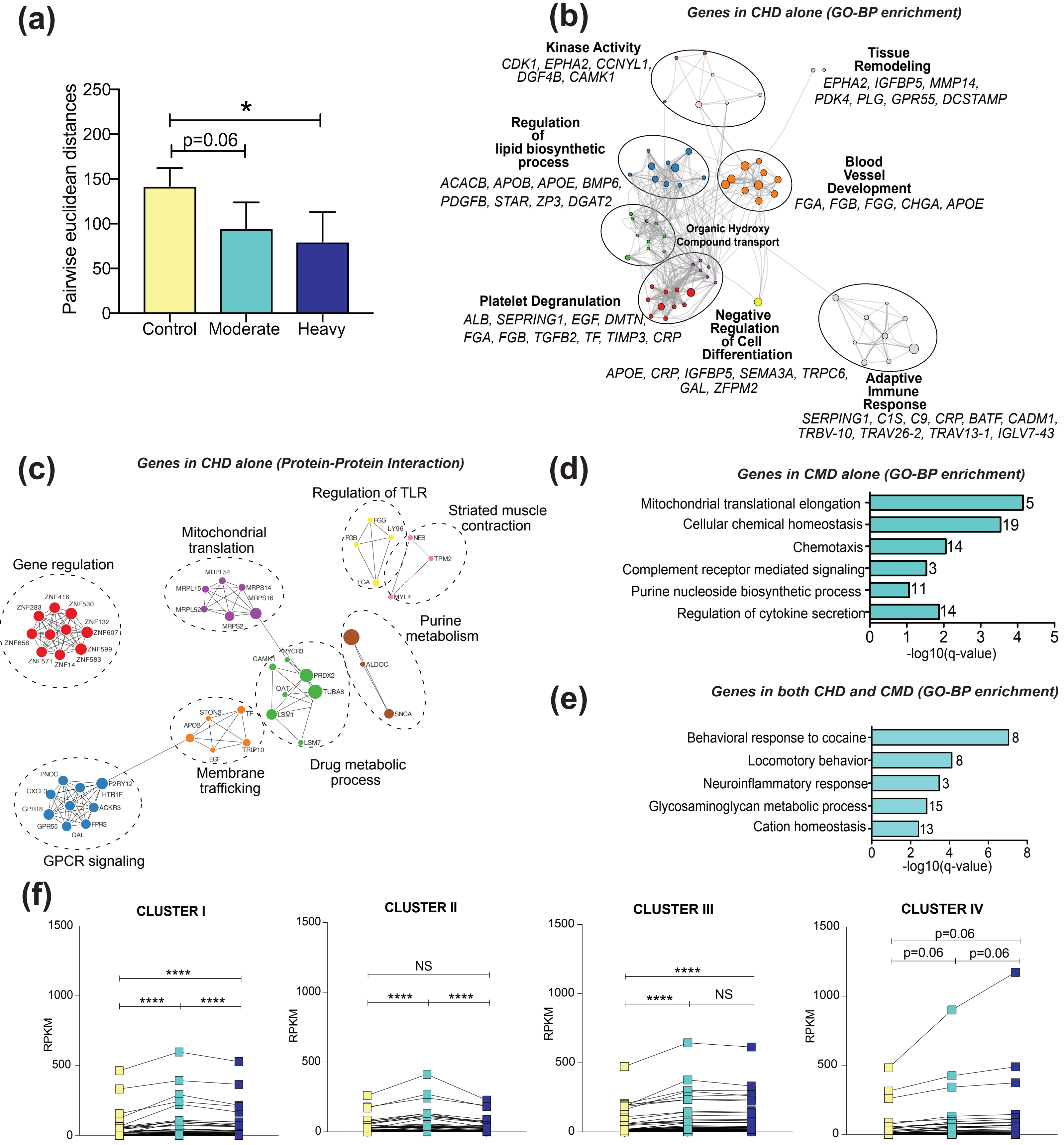

Supplementary Figure 2: Impact of chronic alcohol drinking on PBMC transcriptional profile
(a) Bar graphs of pairwise euclidean distances between samples within each group. Differences between groups were tested using ordinary one-way ANOVA followed by Holm's Sidak multiple hypothesis correction (*- p<0.05) (b) Network depicting functional enrichment of the DEG detected exclusively dysregulated in CHD identified using Metascape. Each colored bubble represents a GO term clustered within a dotted circle with annotations and genes mapping to the terms. Size of the bubble represents number of genes mapping to the GO term. (c) Network depicting protein-protein interactions of all DEG dysregulated with CHD relative to controls identified using Metascape. Each colored bubble represents a protein (DEG) and clusters of proteins enriching to the same GO term are delineated by dotted ellipses. (d) Functional enrichment of the 145 DEG detected exclusively in CMD group relative to controls. Numbers next to the bar represent numbers of genes mapping to each term. (e) Functional enrichment of the 193 DEG detected in common between CMD and CHD relative to controls. Numbers next to the bar represent numbers of genes mapping to each term. (f) Dot plots showing pairwise changes in median RPKMs of genes identified by STEM clustering. (****- p<0.0001, repeated measures ANOVA followed by post-hoc corrections for multiple hypothesis testing).

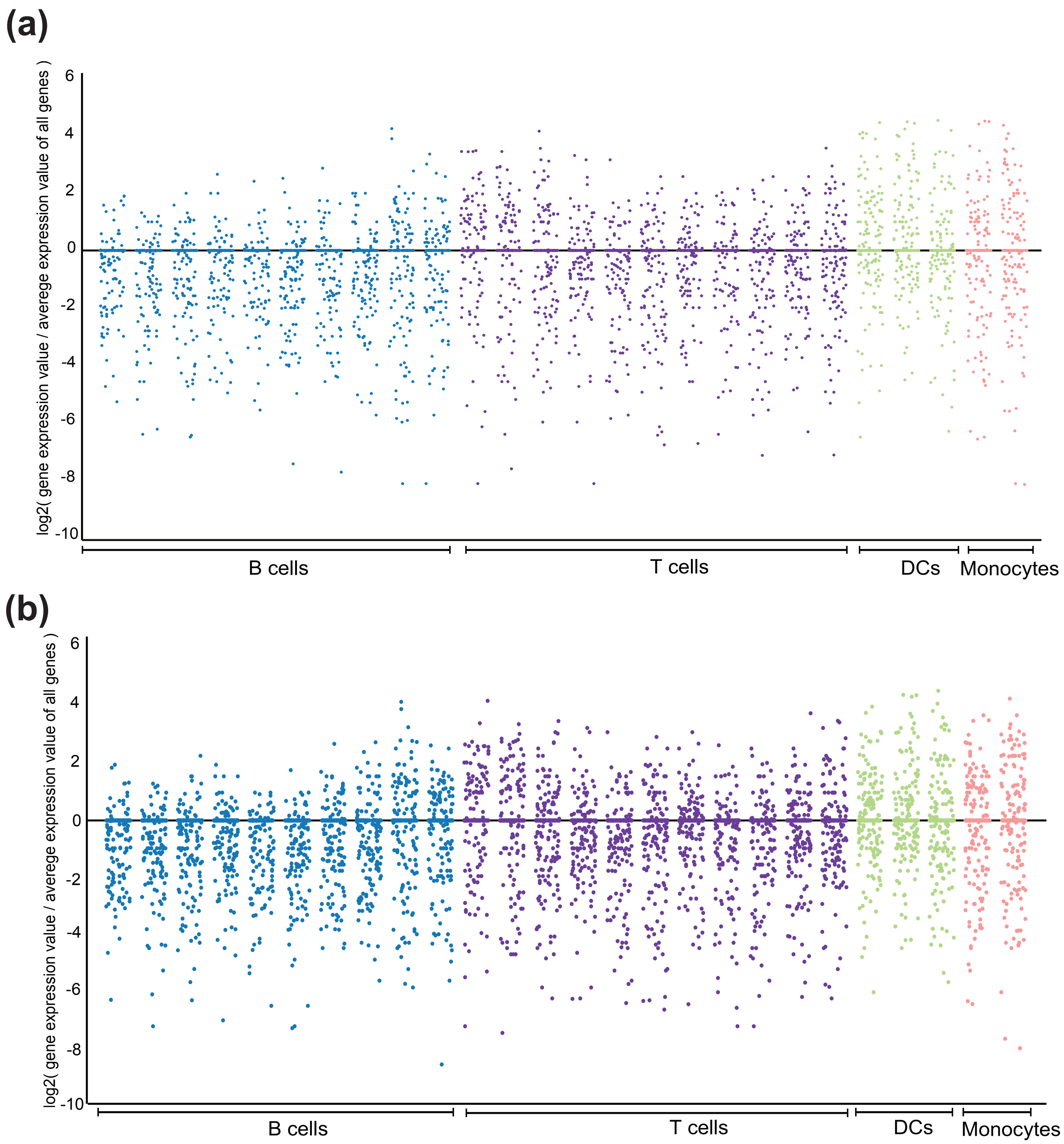

Supplementary Figure 3: Immunological burden of chronic alcohol drinking
Dot plots showing expression profile of DEG detected from (a) CMD and (b) CHD across various immune cell populations from blood as predicted by ImmGen's My GeneSet application.

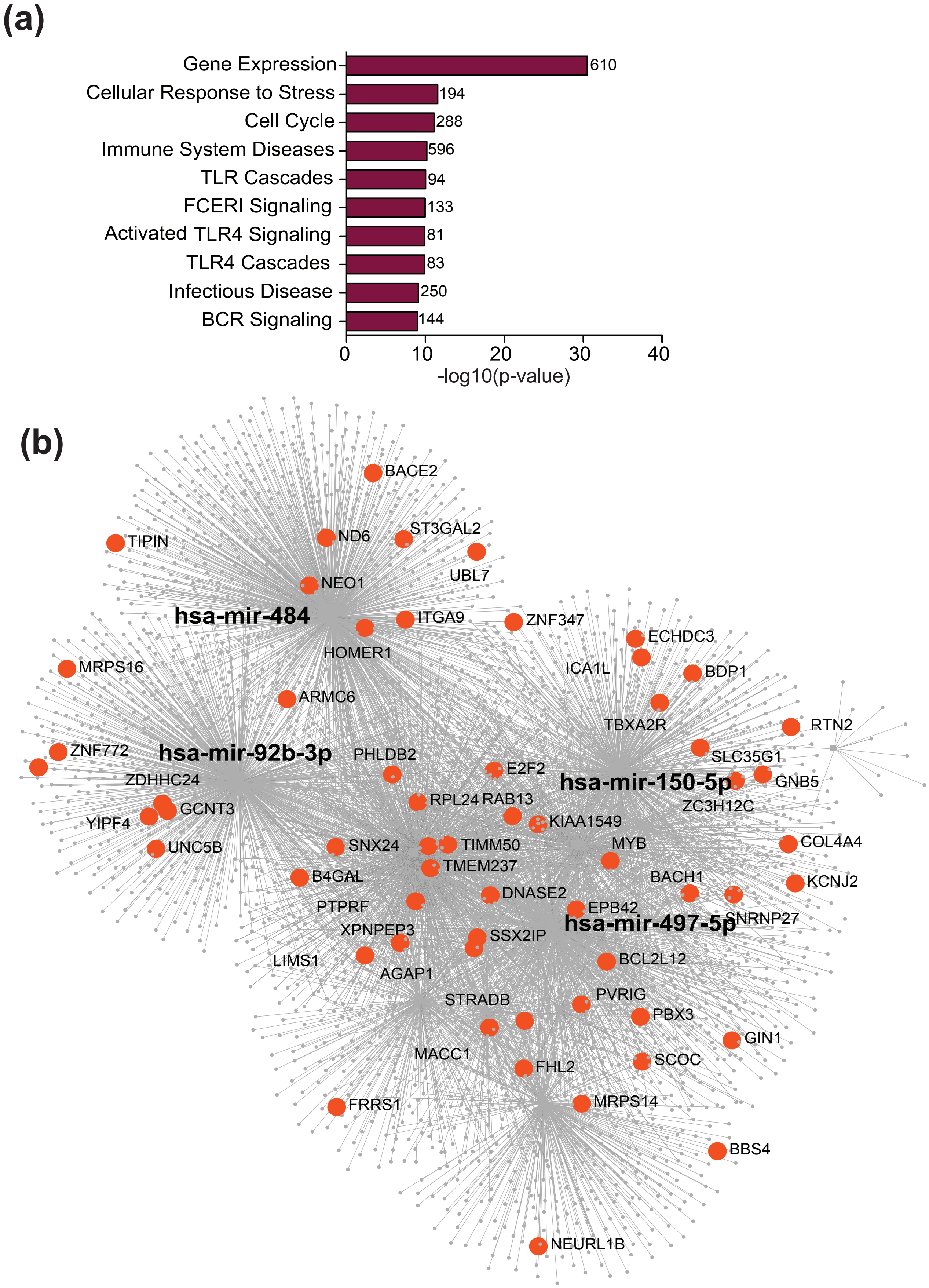

Supplementary Figure 4: Chronic drinking and miRNA profiles in PBMC
(a) Functional enrichment of DEG regulated by the 100 most highly expressed miRNAs in rhesus macaque PBMC. MiRNA expression in control animals was sorted by median RPKM and candidate mRNA partners identified using miRNet. Functional enrichment and p-value calculations were performed using hypergeometric tests. Numbers next to the bars represent number of miRNA interacting partners (gene candidates) that map to the GO term. (b) Network of miRNAs dysregulated with CMD and their validated targets. Only DEG detected in our dataset are highlighted (red circles).

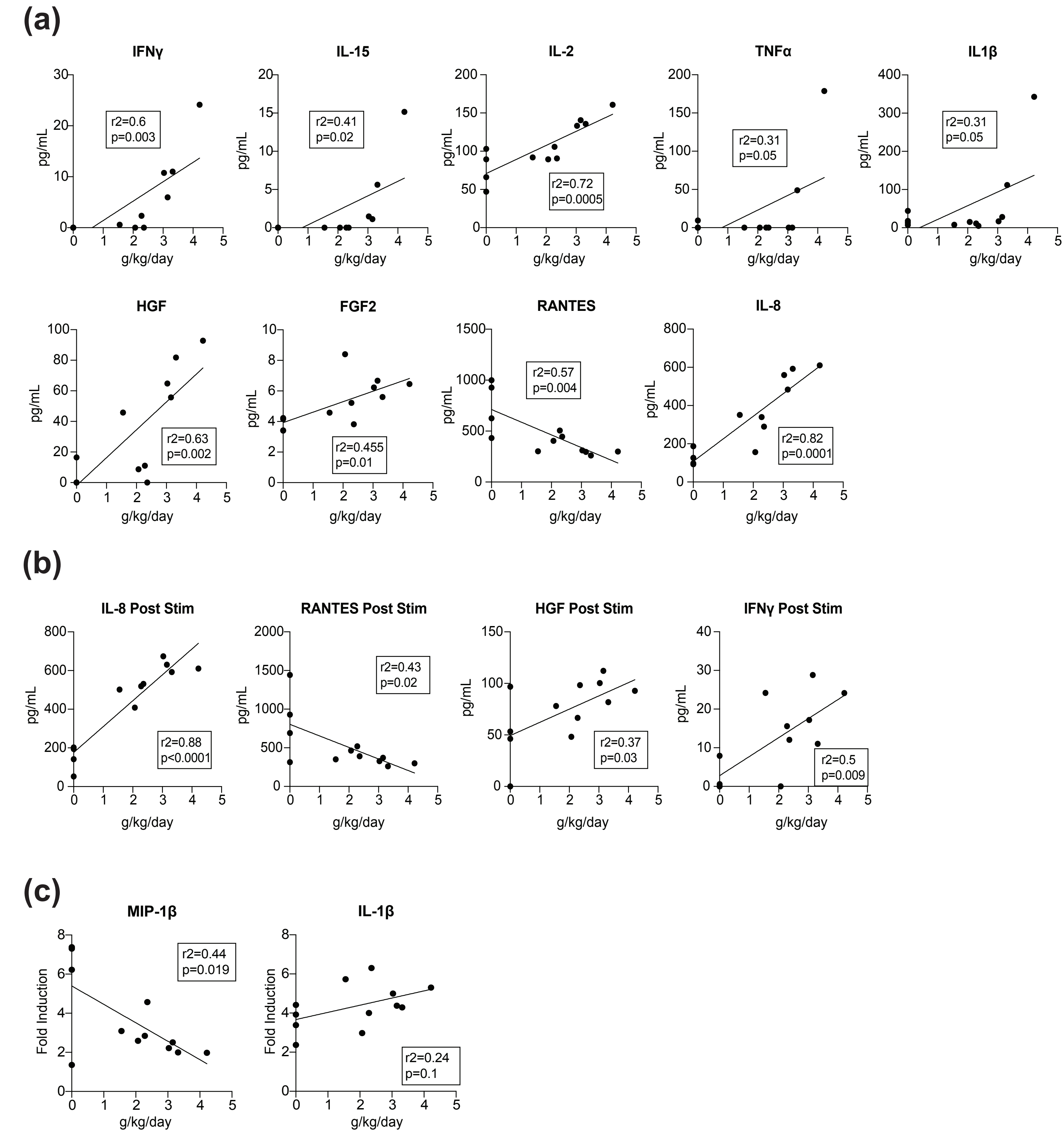

Supplementary Figure 5: Impact of ethanol dose on cytokine and chemokine responses to LPS

(a) Linear regression analyses of analytes that show significant ($p<0.05$) or trending ($p=0.05$) ethanol dose dependent increase or decrease in secreted protein levels in unstimulated PBMC. (b) Regression analysis of proteins that demonstrate dose associated changes post stimulation (c) Regression curves for MIP-1β and IL-1β that show linear associations with dose with respect to fold induction following LPS stimulation.

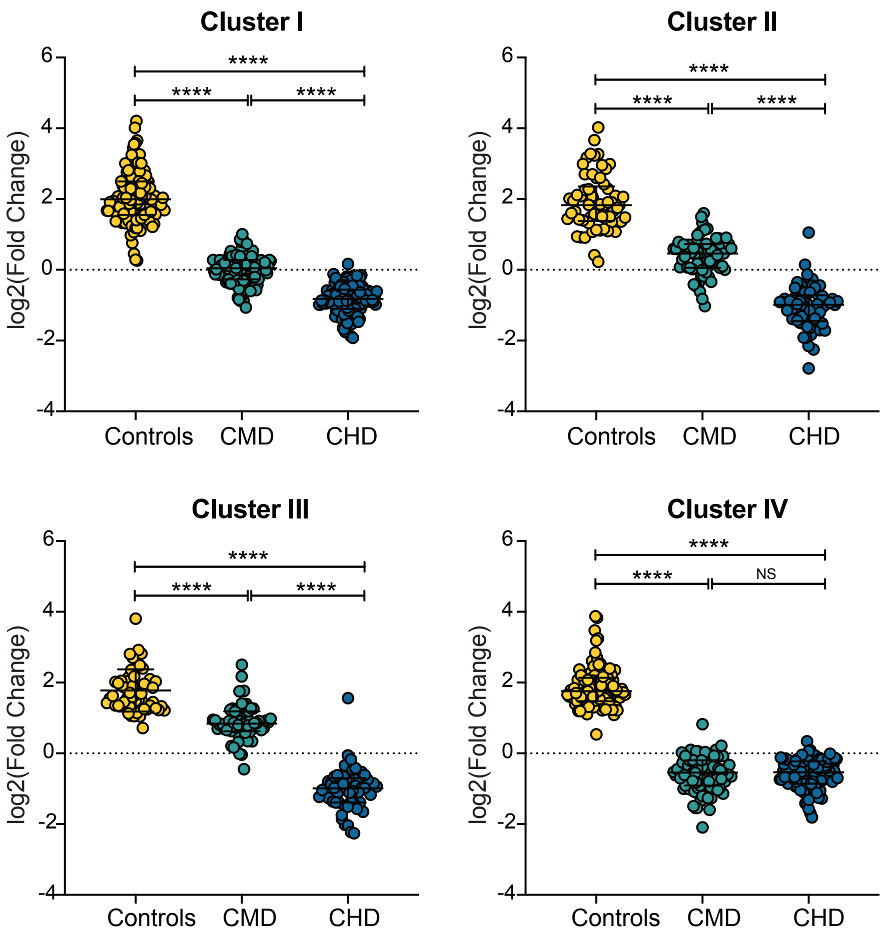

Supplementary Figure 6: Modeling differences in immune responses to LPS.
(A) Dot plots showing pairwise changes in fold changes (log scale) of 325 genes responding to LPS in a dose dependent manner identified by STEM clustering. (****- p<0.0001, repeated measured ANOVA).
